# Protocol for a stepped-wedge, cluster randomized controlled trial of the LifeSpan suicide prevention trial in four communities in New South Wales, Australia

**DOI:** 10.1186/s13063-020-04262-w

**Published:** 2020-04-15

**Authors:** Fiona Shand, Michelle Torok, Nicole Cockayne, Philip J. Batterham, Alison L. Calear, Andrew Mackinnon, Dean Martin, Isabel Zbukvic, Katherine Mok, Nicola Chen, Lauren McGillivray, Matthew Phillips, Henry Cutler, Brian Draper, Grant Sara, Helen Christensen

**Affiliations:** 1 Black Dog Institute, University of New South Wales, Sydney, NSW Australia; 2 Centre for Mental Health Research, Australian National University, Canberra, NSW Australia; 3 Macquarie University Centre for Health Economics, Macquarie University, Sydney, NSW Australia; 4 School of Psychiatry, University of New South Wales, Sydney, NSW Australia; 5 System Information and Analytics Branch, NSW Ministry of Health, Sydney, NSW Australia; 6 Northern Clinical School, Sydney Medical School, University of Sydney, Sydney, NSW Australia

## Abstract

**Background:**

Despite increasing investment in suicide prevention, Australian suicide rates have increased steadily in the past decade. In response to growing evidence for multicomponent intervention models for reducing suicide, the LifeSpan model has been developed as the first multicomponent, evidence-based, system-wide approach to suicide prevention in Australia. The LifeSpan model consists of nine evidence-based strategies. These include indicated, selective and universal interventions which are delivered simultaneously to community and healthcare systems over a 2-year implementation period. This study will evaluate the effectiveness of the LifeSpan model in reducing suicide attempts and suicide deaths in four geographically defined regions in New South Wales, Australia.

**Methods:**

We outline the protocol for a stepped-wedge, cluster randomized controlled trial. Following a 6-month transition phase, the trial sites will move to the 2-year active implementation phase in 4-monthly intervals with evaluation extending a minimum of 24 months after establishment of the full active period. Analysis will be undertaken of the change attributable to the invention across the four sites. The primary outcome for the study is the rate of attempted suicide in the regions involved. Rate of suicide deaths within each site is a secondary outcome.

**Discussion:**

If proven effective, the LifeSpan model for suicide prevention could be more widely delivered in Australian communities, providing a valuable new approach to tackle rising suicide rates. LifeSpan has the potential to significantly contribute to the mental health of Australians by improving help-seeking for suicide, facilitating early detection, and improving aftercare to reduce re-attempts. The findings from this research should also contribute to the evidence base for multilevel suicide prevention programs internationally.

**Trial registration:**

Australia New Zealand Clinical Trials Register, ID: ACTRN12617000457347. Prospectively registered on 28 March 2017. https://www.anzctr.org.au/TrialSearch.aspx#&&conditionCode=&dateOfRegistrationFrom=&interventionDescription=&interventionCodeOperator=OR&primarySponsorType=&gender=&distance=&postcode=&pageSize=20&ageGroup=&recruitmentCountryOperator=OR Protocol Version: 1.0, 31 May 2019.

## Background

Suicidal behavior presents a significant health burden in terms of premature mortality and preventable disability, with more than 3000 deaths per year in Australia [1], compared to 1785 deaths a decade ago [1]. The national suicide rate has increased by 13.4% over the past decade [1]. Large population surveys indicate a similarly stable or increasing pattern for suicide ideation and attempts [2]. There are significant economic costs associated with suicide (e.g., forensic and medical responding, lost productivity), with estimates that suicide deaths alone cost AU$1.7b annually in Australia [3]. The costs associated with self-harm and suicide attempts are likely to increase this amount due to substantial disability and loss of years of healthy life, medical care costs, and secondary distress and productivity impact caused to family members and friends. Despite suicide prevention being a global health priority, the problem does not seem to be improving.

Now, more than ever, government, communities, and organizations around the world are focused on the prevention of suicide, with an increasing number of efforts being funded to take place within, and between, countries. This commitment has led to the development and implementation of a range of interventions aimed at the prevention of suicidal behavior. However, based on a recent review [4] not all initiatives have evidence for preventing suicidal behaviors, and often, less effective strategies, such as public awareness campaigns, may be the most commonly implemented because they may be least resource intensive [5]. Given the scope, severity, and consistency of the suicide problem, it is becomingly increasingly recognized that if an impact is to be made at the population level, evidence-based approaches in suicide prevention are required, and single-intervention approaches are not sufficient. Recent research indicates that even one suicide prevention strategy with the best evidence, implemented in optimal conditions, is likely to have only a small impact on suicide rates [6].

One of the more promising suicide prevention approaches in the past decade has been the development of multicomponent models, which deliver multiple evidence-based strategies, simultaneously, in high-risk geographic regions. This approach has emerged in recognition that no single suicide prevention strategy clearly stands above the others as an effective prevention approach at either the individual or population level [4]. Multicomponent approaches typically combine a range of preventive interventions, spanning from indicated intervention for the highest risk individuals (e.g., people who have attempted suicide) through to universal prevention efforts that apply to the wider community (e.g., reducing access to means, gatekeeper training). Through the combination of universal, selected, and indicated interventions there is increased likelihood of reaching those who are reluctant to seek help, improving early identification, and improving support to high-risk individuals during critical time points for re-attempt. Growing evidence from Europe, the USA, United Kingdom, and Japan indicates that multilevel, multimodal system approaches that target health and community settings are an effective way of reducing the rate of suicide, as compared to traditional silo approaches [7, 8].

In response to the promising evidence for multilevel interventions for reducing suicidal acts (attempts and/or deaths), the LifeSpan model was developed in 2015 [9]. LifeSpan is a large-scale, community-wide trial for suicide prevention that will be implemented in four high-priority regions in New South Wales (NSW), the most populous state of Australia. LifeSpan involves implementing nine evidence-based strategies, simultaneously, for a period of 2 years. Each site has its own local implementation team so that interventions, while maintaining core features for effectiveness, may be tailored to suit the local environment. The four sites are supported by a central implementation team and a research team based at the Black Dog Institute.

The LifeSpan project represents the first scientific trial of a complex, multilevel suicide prevention approach in Australia.

### Primary aim

(i) To examine whether the LifeSpan intervention reduces *suicide attempts* after implementation in the four regions relative to rates and temporal tends established from data covering a period from 2012 until the introduction of LifeSpan in each region

### Secondary aims

(i) To examine whether the LifeSpan intervention reduces *suicide death rates* in the four regions, relative to rates and temporal tends in the pre-intervention period
(ii) To examine change in rates of *suicidal acts* (both suicide deaths and attempts) after introduction of LifeSpan in the four regions, relative to rates and temporal tends in the pre-intervention period
(iii) To examine the change of rates of suicidal acts in the trial sites compared to the rest of NSW where LifeSpan is not being implemented over the period of data collection

### Primary hypothesis

(i) That the suicide attempt rate will be lowered within the intervention regions after the implementation of LifeSpan compared rates and trends established in the pre-implementation period

### Secondary hypotheses

(i) That the rate of suicidal acts will be lowered within the intervention regions after the implementation of LifeSpan compared with rates and trends established in the pre-implementation period
(ii) That the LifeSpan intervention sites will achieve greater reductions in the rate of suicidal acts in the period after the full establishment of the program compared to the remainder of the State (NSW) in which LifeSpan is not being implemented

The LifeSpan research program also has other secondary and tertiary aims and hypotheses, including an examination of the cost-effectiveness of the LifeSpan model, the impact of LifeSpan on help-seeking among the community and at-risk persons, and changes in referral and treatment practices. These aims and outcomes will be described in separate protocols and publications as the data for these questions will be examined in subgroups or different samples.

All study endpoints are reviewed and verified by a research evaluation and monitoring committee.

## Methods

### Study design

**Fig. 1:**
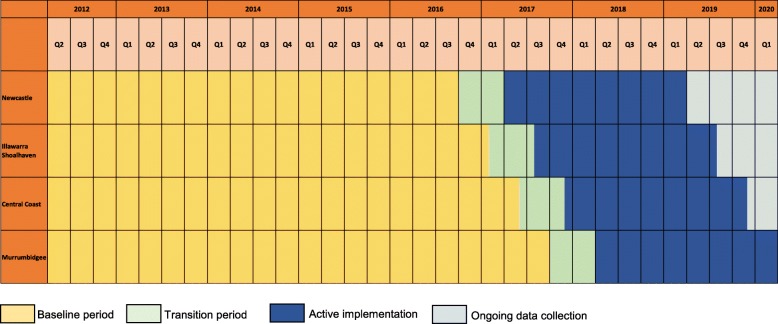
Stepped-wedge trial design including baseline, establishment, active implementation, and data collection periods

The trial uses a community-based, pragmatic, stepped-wedge, cluster randomized design. In this design, the four “clusters” (i.e., trial sites) transition from the control condition to the intervention condition at 4-month intervals, until all clusters are exposed (Fig. 1). Each cluster has an “establishment” or planning period directly prior to moving into the “active intervention” phase of the trial. This is a 6-month period where sites will not be considered fully exposed to the intervention, and as such, this period will be used in the estimation of the effect of LifeSpan. During the establishment phase, site trial coordinators will be employed, suicide collaboratives involving key organizations will be established if they do not already exist, and a local steering committee will be created.

Once a cluster reaches the “active intervention” phase, they have an implementation period of 2 years in which to establish all nine strategies. By the end of this 2-year period, all nine strategies are intended to be operating simultaneously.

The stepped-wedge design was chosen for logistical, political, and economic reasons. The number of clusters required for a randomized controlled trial would have been beyond the scope of the project budget, and it would have been too resource intensive to intervene simultaneously in multiple clusters in a trial of this scale. Moreover, at the time the trial was being considered, there was increasing suicide prevention activity across Australia, which led to concerns about the ability to identify control sites where new suicide prevention activity would not confound the study outcomes. Finally, the investigators were concerned that control regions would find it unacceptable to be allocated to the control condition rather than the intervention condition and be limited in their suicide prevention activities for over 2 years.

### Participants and eligibility criteria

LifeSpan aimed to deliver interventions which were integrated in a healthcare region. Regions were defined as being one or more Local Government Areas (LGAs) which interact meaningfully and fall within the boundaries of a single Local Health District or a Primary Health Network. Lead agencies within NSW were invited to submit a written application as part of an Expression of Interest (EOI) selection process. Lead agencies could be a Local Health District, Primary Health Network, or non-government organization, and needed to demonstrate that they had strong relationships with other key agencies within their region in order to deliver the programs within the LifeSpan model. Applications were assessed by a panel of five individuals holding senior leadership positions in the mental health and suicide prevention sectors, including a National Mental Health Commissioner, the Director of the Mental Health Australia Board, representatives from the NSW Ministry of Health and the NSW Mental Health Commission and NSW Aboriginal Mental Health Workforce Program.

The sites were rated against the following five key selection criteria designed to demonstrate feasibility to implement LifeSpan:

1. Capability and capacity of the lead agency and partnership consortium
2. Proposed model, partnerships and project plan
3. Risk management processes
4. Community engagement and communication plan
5. Indicative budget to support implementation

In addition to the above inclusion criteria, sites needed to be able to yield reliable outcome data on rates of suicide attempts and suicide deaths. Accordingly, sites were required to have a minimum base population of 145,000 persons to allow for sufficient statistical power to detect a primary intervention effect. Archival data on suicide mortality and non-fatal hospital admissions for intentional self-harm were analyzed in a preceding early phase scoping study [10] to allocate LGA geographies into quartiles for suicide mortality and morbidity indicators. LGAs with incidence rates that fell within the top quartile for suicide mortality and/or non-fatal incidents were prioritized for site selection in addition to the EOI inclusion criteria, to ensure that the LifeSpan intervention would be implemented in areas with greatest need for suicide prevention efforts. The following sites were selected as a result of this process (Table 1).

**Table 1:** Trial sites selected to receive the LifeSpan intervention

Site (Local Government Areas within scope)	Lead agency	Site start order
“Newcastle” (Newcastle)	Hunter New England Local Health District	1
“Illawarra Shoalhaven” (Wollongong, Shellharbour, Kiama, Shoalhaven)	COORDINAIRE – the South Eastern New South Wales Primary Health Network	2
“Central Coast” (Gosford, Wyong)	Central Coast Local Health District	3
“Murrumbidgee” (Bland, Cootamundra, Griffith, Hay, Junee, Leeton, Tumut Shire, Wagga Wagga, Young)	Murrumbidgee Primary Health Network	4

### Randomization

After selecting the sites according to the inclusion criteria, the four sites (clusters) were randomized to a start order. Randomization was performed using the “sample” function in R, to generate a random order of numbers between 1 and 4 without replacement. Cluster randomization was undertaken for administrative convenience, and for the ecological validity of providing the intervention at the community level. Randomization was carried out by a statistician not involved in the day-to-day conduct of the trial according to ICH Guidelines.

### Study setting

The LifeSpan model is being delivered into the four distinct geographic regions in NSW described above (Newcastle, Illawarra Shoalhaven, Central Coast, and Murrumbidgee). Geographically defined outcome data were built up to the LGA level from a set of Australian Bureau of Statistics (ABS) 2016 State Suburbs Codes (SSCs). The ABS SSCs will be static for 5 years, meaning that regardless of the boundary variations associated with LGAs during the active trial phase, suicide incidents and delivery of the LifeSpan strategies will remain in-scope.

### Intervention

The LifeSpan intervention consists of nine evidence-based suicide prevention strategies. These strategies include universal strategies designed to reach the entire population (regardless of risk), selective strategies which target subgroups of the general population that are determined to be at risk for suicide and indicated strategies for individuals who are experiencing early signs of suicide crisis or behavior. The implementation of all nine strategies within each region over the 2 years of active intervention (Fig. 1) is managed by site coordinators in collaboration with the LifeSpan central team and relevant organizations or services, with the intention that all are operating simultaneously within a region by the end of Year 2. For some of the strategies, there are multiple programs or resources that are recommended and supported by LifeSpan funding. Trial sites will be required to implement at least one program or resource under each of the nine strategies. The nine interventions are described below, grouped according to universal, selective, and indicated strategies.

### Program components

#### Universal prevention strategies

1. Means’ restriction activities, informed by a detailed suicide profile for each region that identifies hotspots and priority means
2. Training in best-practice Mindframe media reporting guidelines [11] for local journalists and key media representatives
3. School-based suicide prevention programs delivered to all students
4. Public awareness raising of suicide and health promotion in partnership with *R U OK?* [12] and delivered during national suicide prevention awareness week

#### Selective prevention strategies

5. Suicide Prevention Gatekeeper training for the whole community, using the Question, Persuade, Refer (QPR) program [13].
6. Training for frontline responders for upskilling in how to support individuals with suicidal ideation or self-harming behaviors
7. Training in psychosocial treatments for suicide to psychologists and allied health professionals, and implementation of the Collaborative Assessment and Management of Suicidality (CAMS) [14] framework in relevant healthcare settings
8. Capacity building and training in suicide detection for general practitioners (GPs) through face-to-face training programs, and digital tools to assist with early detection and referral

#### Indicated strategies

9. Establishment of aftercare services in sites where such services do not exist, to provide support to individuals following a suicide attempt, and development and dissemination of best practice guidelines for effective crisis and aftercare [15] for emergency departments (EDs)

See Appendix for a comprehensive description of the nine strategies.

### Control condition

As part of the stepped-wedge design, each site contributes pre-intervention or “control” data. Rates suicide attempts and suicide deaths and their temporal trends will be established based on at least a decade of data from 2012, acquired retrospectively, from archival coronial data, ambulance data, hospital separations and ED data. Data will be acquired at a unit (patient) level, with geocoordinates of incident location, so that suicide data can be mapped to each of the four sites.

### Outcomes

#### Primary outcome

The primary outcome of the study will be the rate of treated suicide attempts in the period from full implementation of LifeSpan until March 2020. This rate will be compared to rates and their temporal trend in the pre-intervention period (defined in each site as the period from July 2012 until the start of implementation). There is currently no gold standard dataset for suicide attempt; for this study we will identify self-harm attempts using three key datasets from the Centre for Health Record Linkage, NSW Health, which are outlined in Table 2. Incidents to be included in the linkage will be determined using International Classification of Diseases Australian Modification codes for intentional self-harm (i.e., X60-X84 (intentional self-harm), Y87.0 (sequelae of intentional self-harm)) [16]. Data will be obtained for the period of 2012–2021, where possible, and each incident case will geocoded to a specific incident address using longitude and latitude coordinates. The incident addresses will also be geocoded to a suburb using Australian Bureau of Statistics (ABS) 2016 State Suburb Codes (SSC) so that each case can be assigned to one of the four respective regions. By linking these three datasets, duplicate incidents will be able to be removed and the maximum number of self-harm cases identified. Narrative text fields in all datasets will be reviewed to maximize identification of suicide attempt cases. Waiver of consent is requested for all attempt and mortality data as data aggregation for outcome analyses means that there is a low or negligible risk that individuals will be identifiable.

**Table 2:** Primary and secondary outcome data sources

	Suicide mortality data	Suicide attempt data		
	National Coronial Information System (NCIS)	NSW ambulance data	NSW Admitted Patient Data Collection (APDC)	Emergency Department Data Collection (EDDC)
**Level data to be acquired at**	Individual/point data	Individual/point data	Individual/point data per hospital separation	Individual/point data
**Years data will be acquired for**	Jan 2006 – Dec 2021	Jul 2012 – Jun 2021	Jan 2007 – Jun 2021	Jan 2007 – Jun 2021
**Summary of data**	The NCIS is a data-storage, retrieval, analysis, interpretation, and dissemination system for coronial information. It enables coroners, their staff, public sector agencies, researchers, and other agencies to access coronial data to inform death- and injury-prevention activities	Ambulance data contains a collection of operational information from computer-aided dispatch (CAD), electronic medical records (eMR), and data documented by clinicians through patient healthcare records	APDC records all inpatient separations (discharges, transfers, and deaths) from all public, private, psychiatric, and repatriation hospitals in New South Wales (NSW), as well as public multi-purpose services, private day-procedure centres, and public nursing homes	The EDDC is an administrative data collection detailing presentations to emergency departments at public metropolitan hospitals in NSW. Diagnosis coding is not by trained clinical information manager but by medical, nursing, or clerical personal

#### Secondary outcomes

The secondary outcomes of the study will be changes in the rate of suicide deaths, and changes in suicidal acts (combined deaths and attempts) within each site at 24 months relative to the control baseline period. To assess changes in mortality rates, unit-level mortality data (i.e., individual suicide cases) geocoded to trial-site geographic boundaries or to NSW will be acquired from the National Coronial Information System (NCIS) for all registered suicide deaths in NSW in the period 2006–2021. The method for identifying cases and assigning them to sites is the same as for the primary outcome. To further examine the effects of secular trends on the primary and secondary outcomes, sensitivity analyses will examine whether changes in suicide attempts, acts and deaths were different in trial sites to the rest of NSW. This analysis will assist in controlling for broader social and economic influences on suicide rates.

#### Recruitment and consent

Following the EOI selection process, successful applicants were notified of their acceptance into the LifeSpan trial. To confirm their participation in the trial, a contract describing roles, responsibilities, and key performance indicators was prepared and signed by the Black Dog Institute and the lead agency for each site.

#### Follow-up

Primary and secondary outcome data will be acquired for 12 months post baseline (end of Year 1 of active intervention) and 24 months post baseline (end of Year 2 of active intervention), though the primary point of interest is the latter.

#### Data management

The LifeSpan data environment will be hosted on a stand-alone secure server maintained by the University of New South Wales. Access to this secure server is through unique staff identification accounts and passwords. The raw data within the environment is stored in project-specific folders related to ethical agreements for access. These folders are protected by an Active Directory Rights Management Service (ADRMS) and are only accessible by individuals listed on appropriate ethics agreements. The data team listed on these agreements transforms the raw data into SAS datasets, which are protected by both AD RMS and SAS metadata security permissions. Datasets are then made available to researchers granted access dependent on ethical approval.

#### Sample size and statistical analysis

In the primary outcome analysis, changes in suicide attempt presentation rates per 100,000 population will be tested by comparing the mean annual attempt rate from July 2012 until the implementation date (pre-implementation) with the mean annual attempt rate from the implementation date till the end of the active implementation period (24 months post baseline), combined across all four trial sites. To determine whether the changes in trial-site suicide attempt rates were different to those occurring elsewhere, sensitivity analyses will use the suicide attempt rate for the rest of the NSW population as a background rate. Subsequent analysis will examine changes in treated suicide attempt rates pre and post implementation by individual site. The treated suicide attempt rate will be calculated per 100,000 population using the three linked datasets described for the primary outcome: ambulance data, ED data, and admitted patient data.

To examine changes in suicide deaths, the pre- and post-baseline rates per 100,000 will be calculated using data from the National Coronial Information System. As for the suicide attempt rates, the suicide death rates will be examined for all four trial sites collectively and for each individual site and compared with the background suicide death rates for the remainder of the NSW population in sensitivity analysis.

The alpha level will be set at 0.05 for all analyses.

The mean rate of treated suicide attempt presentations in the period 2005–2013 was approximately 250 per 100,000 per year in the four target sites, based only on hospital admission records. We have previously calculated that the nine strategies could, cumulatively or synergistically, reduce the rate of attempts by up to 30% [6]. However, the magnitude of the effect of a multilevel approach is likely to vary by the extent and length of implementation, the reach of exposure of individuals to multiple strategies, and the existing quality and availability of services and initiatives within communities at baseline. Detecting such change within one trial site relative to another trial site, with 90% power, would require a population within each site of at least 79,000 people. As each trial site has at least 145,000 population, we will be able to detect reductions of approximately 7% in suicide attempt rates within each site.

#### Adverse events

All serious adverse, unexpected, or possibly related events (including data breaches) will be recorded in a case report form and will be reported to the director of the LifeSpan trial and the principle investigator, Professor Helen Christensen. A case report will be submitted to the relevant HREC agency/ies and the relevant data custodian/s. The recommendations from the HREC and data custodians will guide whether the study can continue or be stopped.

## Discussion

This study will use a stepped-wedge, cluster randomized controlled trial design to investigate the impact of the LifeSpan multilevel, multimodal intervention on rates of suicide attempt and suicide death in four geographic regions in NSW. If the program proves efficacious, the LifeSpan model can be implemented in additional high-priority regions, nationally.

This study has a number of limitations which are likely to have an impact on the stated outcomes. Firstly, the scope and intensity of some of the interventions may be difficult to implement within 2 years, particularly resource-intensive interventions (e.g., aftercare services) for which regions have not yet secured funding to establish. Achieving reductions in suicide deaths and attempts will be strongly influenced by our ability to achieve sufficient dose, reach, and sustainability in our implementation efforts. Secondly, it is also likely going to be difficult to monitor fidelity of implementation within a trial of this scale, which is problematic insomuch that the fidelity with which the strategies are implemented is likely to impact effectiveness. Thirdly, the reliance on secondary administrative data for the evaluation may be problematic, as there are data lags in coronial data as well as reporting biases in administrative data which may affect the accuracy of the primary and secondary outcomes. The fourth limitation is raised in relation to the length of the trial, only being 2.5 years’ duration, as this is unlikely to be sufficient time to embed the strategies locally to achieve population-level impact. The length of the trial also raises the risk of dropout of key staff, which may impact how the interventions are delivered on the ground. Relatedly, the interventions (e.g., school-based programs) may have a delayed effect that is not captured within the trial period. Fifth, while our analysis will account for the influence of some broader social and economic factors on suicide rates, it may not account for local changes (e.g., local changes in employment conditions, natural disasters). To address this, we are documenting significant local events and changes that may influence suicide rates and that may help to understand some regional variations in outcomes. Finally, given the complexity of the model, it will not be possible to measure the potential synergistic interactions between interventions and relate this to magnitude of change in suicide attempts at an area level or population level within the trial sites.

Some of the strengths of this study are as follows: this is the first suicide prevention trial of its kind in Australia; the emphasis on the use of evidence-based and multiple strategies to reach everyone in the community, instead of targeting a specific strategy or problem area. The results of this study are likely to have impact for research and policy, internationally, given the innovation of the LifeSpan model and the quality of the data that we will acquire. Achieving implementation at-scale and ensuring sustainability will be immensely challenging. Yet, understanding implementation and sustainability is key to the success of multilevel models such as LifeSpan, and what is learned here is likely to have implications for other highly complex problems. In addition to measuring outcomes, we have commissioned research to examine implementation and sustainability. Given the scale and complexity of this work, the comprehensive findings of this research will be published separately, and will also be referenced in discussing the main outcomes of the LifeSpan trial.

## Trial status

The selection process for trial sites was undertaken in May and June 2016, and the selection process was completed in July 2016. The trial sites were randomized and allocated in July 2016. Archival data for the evaluation will be collected from 1 April 2012, and the last data collection (indicating end of recruitment) will be acquired in August 2021 (for suicide attempt) and April 2022 for coronial data (based on current average time lags).

## Data Availability

The data that support the findings of this study are available from the National Coronial Information System, NSW Health, and NSW Ambulance, but restrictions apply to the availability of these data, which were used under license for the current study, and so are not publicly available. Data may, however, be available from the authors upon reasonable request and with permission of the above-named data custodians.

## Appendix

### The intervention components

#### Universal prevention strategies

1. *Means’ restriction.* A suicide audit report will be provided to the site-lead agencies and suicide prevention collaboratives of each site during the transition phase. This report will include the analysis of coronial, hospitalization, and ambulance data to establish the demographic, socioeconomic, and suicide means suicide risk profiles for each site. It will also provide recommendations for tailored means’ restrictions’ strategies against identified priority populations. The audit will include a detailed appendix of evidence-based strategies to reduce access to, or lethality of, means, which is intended as a practical strategy to help sites decide what specific means’ restriction strategies should be implemented in their local region
2. *Media guidelines.* Local media representatives will receive group-based face-to-face media training delivered by Mindframe Australia, aimed at reducing dangerous reporting of suicide, and increasing responsible and respectful coverage of mental health and suicide in the media. The target audience is local journalists, suicide prevention multiagency group members, organizational leaders, and local leaders who might be asked to comment on suicide by media and who are involved in community support after suicide. The training will be a 2-day, face-to-face group program delivered in Year 1 of active implementation at each site, with a half-day refresher program delivered in Year 2
3. *School-based suicide prevention.* Year-9 high-school students in all public, independent, and Catholic high schools within the four sites will be offered an evidence-based, universal suicide prevention program designed to reduce stigma, increase help-seeking behaviors and resilience, and reduce suicidal ideation and behaviors. The Youth Aware of Mental Health (YAM) program will be delivered over the 2-year duration of the LifeSpan trial, meaning two consecutive cohorts of Year-9 students will receive YAM. The YAM program was developed by researchers and clinicians from the Karolinska Institute in Sweden and Cornell University in the USA. Its primary aim is to raise awareness about suicide risk and the factors that may protect against suicidality. YAM consists of five sessions delivered over a 3-week period, and involve a mix of direct instruction, reading material, visual information and interactive workshops where young people are encouraged to role-play skills within a supervised environment
4. *Public awareness.* Centrally developed resources and materials with key messaging will be disseminated to the general public within each of the sites through a series of awareness-raising community events, in partnership with “R U OK” campaign providers. The LifeSpan resources and messages will be designed to engage the community in suicide prevention, with a call to action; for example, to complete gatekeeper training; sign up to be a YAM helper; to reduce stigma; and to increase identification and referral of individuals with suicidal ideation or self-harming behaviors. Major awareness-raising events will be held in September in Year 1 and Year 2, during the national suicide prevention awareness week

#### Selective strategies

5. *Gatekeeper training.* Question, Persuade, Refer (QPR) training will be provided to all community members in each of the four sites, to skill them in being able to recognize and respond positively to someone exhibiting suicide warning signs and behaviors, including providing appropriate referral information. This will be a brief (60-min) training program, that individuals will sign up to, and then they will be able to access their licence for up to 3 years, to refresh their gatekeeper skills. The digital QPR program will be available at all times throughout the active implementation period in each site, and it will be advertised through the public awareness strategy to increase uptake in the community
6. *Frontline responder training.* First responders (police, paramedics, and ED staff) will receive face-to-face and online training to support individuals with suicidal ideation or self-harming behaviors. Training will be provided to first responders in each of the four sites. Where training already exists, such as the NSW Police Mental Health Intervention Training (MHIT) program, support will be offered to ensure that the program is meeting local needs. The local implementation teams will identify key decision-makers within each sector to work with them in implementing training. The total number of staff trained will vary by sector and site
7. *Psychosocial treatments.* A guide to evidence-based psychological and pharmacological treatments for the care of individuals with suicidal ideation and/or behaviors will be provided to privately and publicly employed psychologists, psychiatrists, and other mental health professionals at each site. The Advanced Suicide Prevention Training program, designed and facilitated by the Black Dog Institute, will be provided for these professionals, to inform appropriate assessment of suicidal ideation and/or behaviors. This 6-h face-to-face group program trains participants to undertake a suicide risk assessment effectively; develop a collaborative safety plan; implement a team approach to treatment planning; provide effective management following a suicide attempt; and respond to the needs of people bereaved by suicide. This training will be supplemented by information on local referral pathways, delivered by a local subject-matter expert and program champion. We aim to train 200 mental health professionals at each of the four sites. The Collaborative Assessment and Management of Suicidality (CAMS) framework will be implemented in relevant healthcare settings in each site, with mental health teams selected to participate according to their role in providing treatment to people at risk of suicide. CAMS is an evidence-based approach to managing suicide risk [17, 18] and its implementation entails structured training and implementation support

8. *General practitioner (GP) capacity building and training.* General practitioners will receive training and practice support to identify and refer individuals with suicidal ideation or self-harming behaviors. The Advanced Suicide Prevention Training program, designed and facilitated by the Black Dog Institute, will be provided to general practitioners. This 6-h face-to-face group-based program trains participants to undertake a suicide risk assessment effectively; develop a collaborative safety plan; implement a team approach to treatment planning; provide effective management following a suicide attempt; and respond to the needs of people bereaved by suicide. In addition, general practices will be provided with training by the Black Dog Institute-developed “StepCare” program. StepCare is designed to digitally screen patients for depression, anxiety, substance use, and suicidal thoughts while they wait for their appointment. GPs will be provided with detailed immediate feedback, verbal scripts and recommendations for the patient via the practice management software. Training for the general practice staff in StepCare will come from a trainer located within the Primary Health Network, who will have received “train-the-trainer” from the Black Dog Institute StepCare coordinator. We aim to train and/or provide StepCare access to 50–60 general practitioners per site

#### Indicated strategies

9. *Aftercare and crisis care following a suicide attempt.* The reform of care in the emergency department (ED) and aftercare will be ongoing through the 2-year implementation phase of LifeSpan. A set of best-practice guidelines for effective crisis and aftercare has been developed [15] and will be implemented in collaboration with EDs in each of the four sites. The guidelines recommend that all individuals presenting to the ED for a suicidal crisis receive a comprehensive psychosocial assessment, regardless of their perceived level of risk. This includes clear communication to the patient about the role of the psychosocial assessment, with an emphasis on the relational aspects of the assessment. Each site will also commission an aftercare support service that includes assertive outreach, solution-focused counseling, support to adhere to treatment, and where possible, continuity of contact with the same staff member for up to 12 weeks

## Abbreviations

ABS: Australian Bureau of Statistics
ADRMS: Active Directory Rights Management Service
APDC: Admitted Patient Data Collection
CAMS: Collaborative Assessment and Management of Suicidality
ED: Emergency department
EDDC: Emergency Department Data Collection
EOI: Expression of Interest
GP: General practitioner
HREC: Human Research Ethics Committee
LGA: Local Government Area
MHIT: Mental Health Intervention Training
NCIS: National Coronial Information System
NSW: New South Wales
QPR: Question, Persuade, Refer
SAS: Statistical Analysis System
SSC: State Suburbs Code
USA: United States of America
YAM: Youth Aware of Mental Health

## References

[1] Australian Bureau of Statistics (2018). Causes of Death, Australia, 2017 (cat. no. 3303.0).

[2] Kessler RC, Berglund P, Borges G, Nock M, Wang PS (2005). Trends in suicide ideation, plans, gestures, and attempts in the United States, 1990-1992 to 2001-2003. Jama..

[3] KPMG Health Economics. The economic cost of suicide in Australia. Australia; 2013.

[4] Zalsman G, Hawton K, Wasserman D, van Heeringen K, Arensman E, Sarchiapone M (2016). Suicide prevention strategies revisited: 10-year systematic review. Lancet Psychiatry.

[5] Torok M, Calear A, Shand F, Christensen H (2017). A systematic review of mass media campaigns for suicide prevention: understanding their efficacy and the mechanisms needed for successful behavioral and literacy change. Suicide Life Threat Behav.

[6] Krysinska K, Batterham PJ, Tye M, Shand F, Calear AL, Cockayne N (2016). Best strategies for reducing the suicide rate in Australia. Austr N Z J Psychiatry.

[7] Hegerl U, Wittmann M, Arensman E, Van Audenhove C, Bouleau JH, Van Der Feltz-Cornelis C (2008). The “European Alliance Against Depression (EAAD)”: a multifaceted, community-based action programme against depression and suicidality. World J Biol Psychiatry.

[8] Knox KL, Litts DA, Talcott GW, Feig JC, Caine ED (2003). Risk of suicide and related adverse outcomes after exposure to a suicide prevention programme in the US Air Force: cohort study. BMJ.

[9] Black Dog Institute. Torok M, Shand F, Krysinska K, Batterham P, Konings P, Calear A, Cockayne N, Christensen H. A systems approach to suicide prevention: implementation plan. Sydney; 2015.

[10] Torok M, Konings P, Batterham PJ, Christensen H (2017). Spatial clustering of fatal, and non-fatal, suicide in new South Wales, Australia: implications for evidence-based prevention. BMC Psychiatry.

[11] Mindframe. Resources about suicide, mental ill-health, alcohol and other drugs—Mindframe. Everymind. Retrieved from https://mindframe.org.au/ Accessed June 2019.

[12] OK? RU. Suicide prevention | R U OK? R U OK? Retrieved from https://www.ruok.org.au/; Accessed June 2019.

[13] QPR. Practical and Proven Suicide Prevention Training QPR Institute. QPR Institute. Retrieved from https://qprinstitute.com/ Accessed June 2019.

[14] Jobes DA (2012). The Collaborative Assessment and Management of Suicidality (CAMS): an evolving evidence-based clinical approach to suicidal risk. Suicide Life Threat Behav..

[15] Hill NTM, Shand F, Torok M, Halliday L, Reavley NJ (2019). Development of best practice guidelines for suicide-related crisis response and aftercare in the emergency department or other acute settings: a Delphi expert consensus study. BMC Psychiatry.

[16] World Health Organization. The ICD-10 classification of mental and behavioural disorders : clinical descriptions and diagnostic guidelines. Geneva; 1992.

[17] Andreasson K, Krogh J, Wenneberg C, Jessen HKL, Krakauer K, Gluud C, et al. Effectiveness of Dialectical Behavior Therapy versus Collaborative Assessment and Management of suicidality treatment for reduction of self-harm in adults with Bordeline Personality Traits and Disorder—A randomized observer-blinded clinical trial. 2016;33(6):520–30. 10.1002/da.22472 26854478

[18] Comtois KA, Jobes DA, O’Connor S, Atkins DC, Janis K, Chessen EC (2011). Collaborative Assessment and Management of Suicidality (CAMS): feasibility trial for next-day appointment services. Depress Anxiety.

